# Emerging zoonoses.

**DOI:** 10.3201/eid0403.980328

**Published:** 1998

**Authors:** J Childs, R E Shope, D Fish, F X Meslin, C J Peters, K Johnson, E Debess, D Dennis, S Jenkins

**Affiliations:** Centers for Disease Control and Prevention, Atlanta, Georgia, USA.


### Emerging Zoonoses

**James Childs,* Robert E. Shope,† Durland Fish,‡ Francois X. Meslin,§ Clarence J. Peters,* Karl Johnson,¶ Emilio Debess,# David Dennis,* and Suzanne Jenkins****

*Centers for Disease Control and Prevention, Atlanta, Georgia, USA; †University of Texas Medical Branch at Galveston, Galveston, Texas, USA; ‡Yale School of Medicine, New Haven, Connecticut, USA; §World Health Organization, Geneva, Switzerland; ¶University of New Mexico, Albuquerque, New Mexico, USA; #National Association of Public Health Veterinarians; and **Council of State and Territorial Epidemiologists

Zoonotic pathogens cause infections in animals and are also transmissible to humans; knowledge of the extrahuman reservoirs of these pathogens is thus essential for understanding the epidemiology and potential control of human disease. Zoonotic diseases are typically endemic and occur in natural foci. However, ecologic change and meteorologic or climatic events can promote epidemic expansion of host and geographic range. For practical reasons, surveillance of zoonotic agents too often relies on the identification of human cases. Surveillance in natural hosts may be difficult because of the ecologic complexity of zoonoses; multidisciplinary teams of ecologists, mammalogists, ornithologists, and entomologists, as well as physicians and epidemiologists, may be required for successful investigations. A recent trend in studying zoonoses that have strong environmental correlates includes geographers and mathematical modelers, who integrate satellite remote sensing and geographic information systems to predict outbreaks. Understanding extrahuman life cycles and predicting zoonotic disease outbreaks may permit control activities targeted at several points in the cycle of pathogen maintenance before human infection begins. These control efforts are important because most zoonoses are not amenable to eradication, except perhaps those in areas where animal reservoirs are targeted for vaccination, e.g., fox rabies in Europe.

In the United States, tick-borne zoonoses have emerged at the relatively constant rate of one per decade over the past 100 years. However, the incidence of human tick-borne disease has increased exponentially over the past two decades—primarily because of ecologic change caused by reforestation. Large-scale reforestation of the northeastern coastal states since the early part of this century precipitated a natural succession of ecologic changes that included increased deer density, expansion of the natural range of the deer-dependent tick *Ixodes scapularis*, and increased transmission rates of tick-borne pathogens. *I. scapularis* is a competent vector of at least four enzootic tick-borne pathogens *(Borrelia burgdorferi, Babesia microti, Ehrlichia phagocytophila*, and a Powassanlike encephalitis virus). Because of its anthropophilic nature, *I. scapularis* is also an excellent bridge vector for transmission of these pathogens to humans. This dramatic expansion in the distribution of *I. scapularis* in the northeastern United States has caused the current epidemic of Lyme disease and has increased the range of human babesiosis in New England. However, the recent emergence of human granulocytic ehrlichiosis resulted from the recognition of human cases caused by a zoonosis already well established within *I. scapularis* populations. As *I. scapularis* continues to expand its range bringing more people in contact with novel enzootic tick-borne pathogens, additional tick-borne diseases may emerge as new public health threats.

Since the unprecedented impact of bovine spongiform encephalopathy (BSE) and new variant Creutzfeldt-Jakob disease (nvCJD) on animal health and national politics and economies, this new zoonosis has prompted many questions in the field of foodborne disease control and prevention. BSE and nvCJD, caused by an unconventional agent, the nature of which remains controversial, are invariably fatal. The threat to human health is compounded because the causative agent is resistant to conventional physical and chemical methods of decontamination and cannot be fully inactivated by any of the current food technologies. A preclinical diagnostic test remains elusive. Traditional food safety programs cannot prevent infection to consumers once the agent has entered the food chain. New and reemerging conventional or unconventional foodborne pathogens of animal origin must be better addressed, and food safety programs with emphasis on the preharvest and harvest food stages must be developed. Control is best achieved at the feed preparation and farm level and at the harvest stage. Consumer health takes precedence over market concerns, and when data are incomplete, a conservative response is warranted until the risk can be accurately assessed.

Diseases of humans caused by rodent-borne viruses in the families *Bunyaviridae* and *Arenaviridae* include the newly recognized hantavirus pulmonary syndrome and the South American hemorrhagic fevers. Many of these diseases present control challenges—because vaccines may not be developed, because of characteristics of the exposed population, or because control of rodent reservoirs in the affected areas is impractical or unachievable. Many of these diseases may be increasing in frequency as humans modify forest and natural savannah environments for agriculture, inadvertently promoting human-rodent contact and increasing the number of suitable habitats used by rodents, which are habitat generalists and also virus reservoirs. Serious gaps in our understanding of the natural history of these viruses and their hosts limit a targeted intervention. The prevalence of virus infection in rodent populations may be less important than the absolute number and demographic characteristics of infected mice. A single, newly infected, subadult mouse may shed in its urine and feces the high quantities of virus needed to infect a person by the aerosol route. However, effective maintenance of virus may hinge on persistent infections in older, dominant, male rodents that survive over extended periods and have the highest prevalence of infection but only shed sufficient virus in their saliva to perpetuate rodent-to-rodent transmission through intraspecific aggressive encounters. Recent developments in remote sensing and geographic information systems, coupled with longitudinal studies of virus activity and rodent population dynamics, hold promise for developing models predictive of when and where outbreaks of rodent-borne zoonoses could occur.

Surveillance of the unknown appears to be a thankless task, and it is probable that we will learn of an “Andromeda” event after an urban population is struck, although the agent is most likely to arise in a rural, tropical setting. The health and safety of future generations may depend on our ability to rapidly detect, monitor, and control disease caused by novel zoonotic agents. Uniform surveillance definitions, reliable specimen collection, shipping and handling, and means for rapid communication will be critical.

